# Co-targeting of IGF1R/mTOR pathway by miR-497 and miR-99a impairs hepatocellular carcinoma development

**DOI:** 10.18632/oncotarget.18207

**Published:** 2017-05-24

**Authors:** Henghui Cheng, Jin Xue, Shouhua Yang, Yaobin Chen, Yu Wang, Yuanli Zhu, Xiaoyan Wang, Dong Kuang, Qiurong Ruan, Yaqi Duan, Guoping Wang

**Affiliations:** ^1^ Institute of Pathology, Tongji Hospital, Tongji Medical College, Huazhong University of Science and Technology, Wuhan 430030, P. R. China; ^2^ Department of Pathology, School of Basic Medicine, Tongji Medical College, Huazhong University of Science and Technology, Wuhan 430030, P. R. China; ^3^ Department of Obstetrics and Gynecology, Union Hospital, Tongji Medical College, Huazhong University of Science and Technology, Wuhan 430030, P. R. China

**Keywords:** hepatocellular carcinoma, miR-497, miR-99a, IGF1R, mTOR

## Abstract

Persistent activation of IGF1R/mTOR signaling pathway plays crucial role in the development of hepatocellular carcinoma (HCC). Therefore, our goal was to elucidate microRNAs (miRNAs) targeting IGF1R/mTOR and the therapeutic potential of single or dual miRNA on HCC development. In this study, we found that miR-497 and miR-99a that target the 3′-UTR of both IGF1R and mTOR were down-regulated in HCC human tissues and cell lines. Functional assay revealed that ectopic expression of miR-497 or miR-99a in HCC cells resulted in a significant inhibition on tumor growth and invasiveness *in vitro* and tumor development *in vivo* via repressing the expression of IGF1R and mTOR. Such inhibitory effect on tumor growth is reversed by application of IGF1 ((IGF1R ligand) or MHY1485 (mTOR agonist) *in vitro*. Furthermore, we found that simultaneous over-expression of both miR-497 and miR-99a exhibited much stronger inhibitory effects on tumor growth than their individual effect, which is still correlated with significantly stronger repression of IGF1R and mTOR. Overall, our results suggest that miR-497 and miR-99a both function as tumor-suppressive miRNAs by suppressing IGF1R/mTOR signaling pathway. The synergistic actions of these two miRNAs partly correlated with IGF1R and mTOR levels, which may represent new strategies for the molecular treatment of HCC.

## INTRODUCTION

Hepatocellular carcinoma (HCC) is the most common primary liver malignancy in adults [1, 2]. Surgery is the gold-standard treatment and radiofrequency ablation or transarterial chemoembolization are essential complements [3]. But in advanced HCC, the treatment options are still limited and ineffective. Traditional systemic therapy with cytotoxic drugs always has low objective response rates (typically <10%) [4]. Recently, advances in new therapeutic methods, such as biotherapy, especially the molecule targeted therapy, were reported. For example, sorafenib, a tyrosine kinase inhibitor, has become a standard treatment for advanced HCC in recent years [3, 4]. Unfortunately, it prolongs median survival by little more than one year [5]. Therefore, more studies need to be conducted on the genetic alteration of HCC.

HCC development is a very complex biological process. Up to now, some molecular signaling pathways have been found to be involved in cancer initiation and progression, such as Wnt/β-catenin, AKT, hedgehog, MAPK, ERK signaling pathways, and IGF1R/mTOR signaling pathway [6–8]. In patients with HCC and human hepatoma cell lines, overexpression of IGF-IR and mTOR were observed [8–10]. Studies carried out with animal models suggested that hyperactivation of IGF1R/mTOR signaling pathway may promote hepatocarcinogenesis, while blocking this pathway showed promising reduction of HCC tumor growth [9, 10]. Though IGF1R/mTOR signaling pathway is important in development of HCC, the regulatory mechanism of this pathway is not thoroughly understood yet.

Recent studies suggested that IGF1R/mTOR signaling pathway could be regulated by microRNAs (miRNAs) [11–13], a class of single-stranded non-coding RNA molecules which involved in a series of important cellular activities by binding to the 3’UTR of mRNA [14, 15]. Based on microarray and high throughput qRT-PCR techniques, different microRNA expression profiles of HCC and normal tissue are identified. Some of them have been confirmed to target genes involved in some important signaling pathways associated with HCC development. MiR-149, miR-34a, miR-122 and miR-17-5p were down-regulated in HCC, and their direct targets had also been demonstrated to be involved in AKT [16], ERK [17], Wnt/β-catenin [18], or MAPK signaling pathways [19]. Besides, miR-133a, miR-145 and miR-99a were reported to be involved in the initiation and progression of HCC by targeting the expression of IGF1R or mTOR, which shed light on the precise regulation of IGF1R/mTOR signaling in hepatocarcinogenesis [11–13]. However, it is not known whether miRNAs can cooperatively regulate this signaling process.

In the present study, we found that the expression of miR-497 and miR-99a were significantly decreased in human HCC tissues and cell lines. By targeting both IGF1R and mTOR, miR-497 or miR-99a exerted remarkable tumor suppressive function *in vivo* and *in vitro*. Further cotransfection of miR-497 and miR-99a exhibited much stronger inhibition on HCC cell proliferation and xenograft tumor growth. The synergistic effect can be explained by the further inhibition of target gene IGF1R and mTOR. Therefore, miR-497 and miR-99a may play important suppressive effects on tumor growth controlled by IGF1R/mTOR signaling pathway and would be both biological and clinical targets for future HCC research. Targeting dual miRNAs might be a preferred strategy for cancer therapy.

## RESULTS

### IGF1R and mTOR were the target genes of miR-497/miR-99a

As IGF1R/mTOR signaling pathway plays an important role in the development of HCC, we try to explore miRNAs that can target both IGF1R and mTOR. We searched miRNAs predicted to target both IGF1R and mTOR in 10 popular miRNA databases (TargetScan, miRWalk, et al), and 9 miRNAs were identified in half of these databases (Supplementary Table 1). Among these miRNAs only miR-99a and miR-497 were found to be significantly down-regulated in HCC according to two published microarray-based high throughput assessment (NCBI/GEO/GSE21362, NCBI/GEO/GSE36915, P value < 0.001, Supplementary Figure 1). Therefore our study focused on the functional role of miR-99a and miR-497 in HCC.

**Figure 1:**
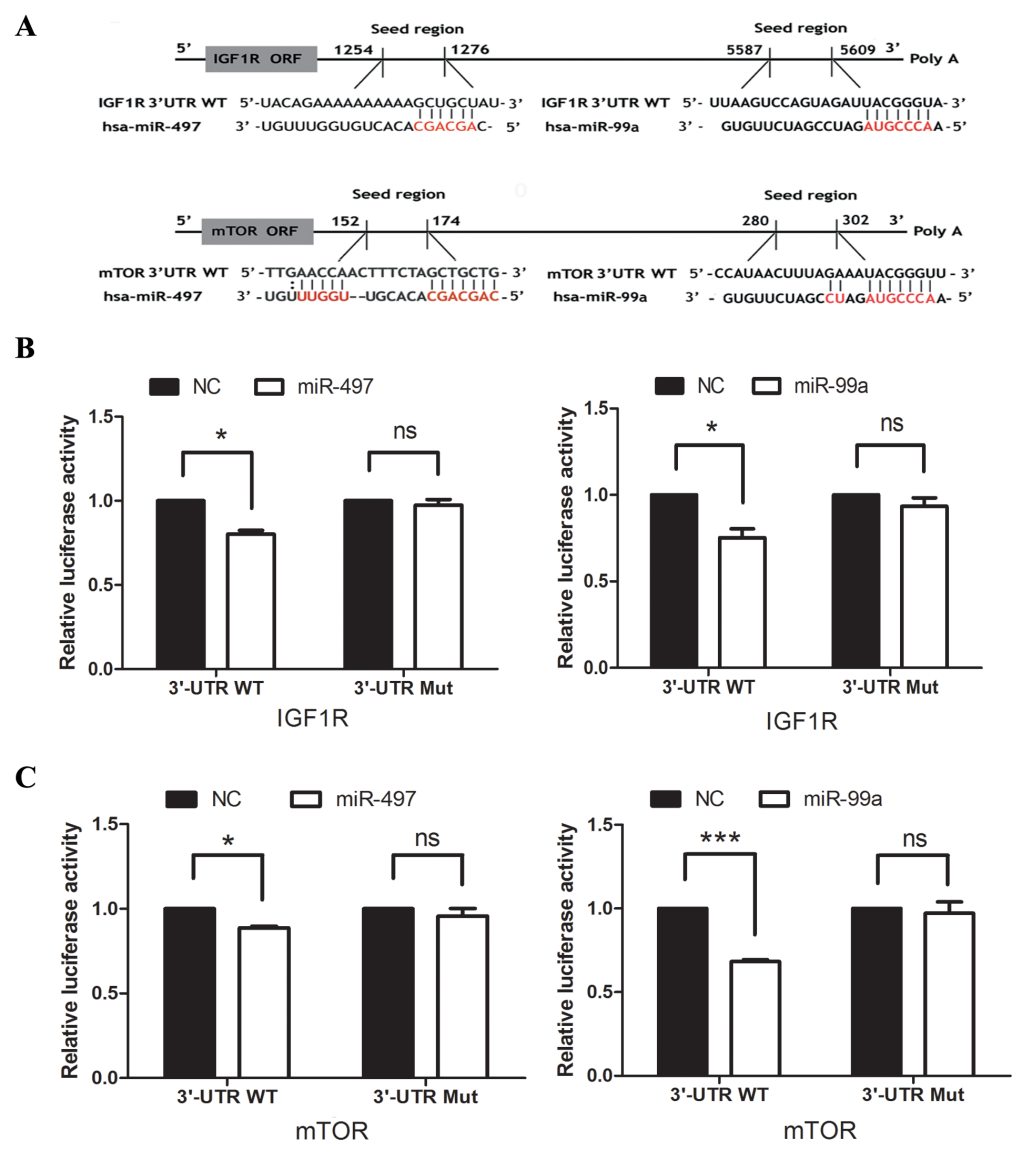
IGF1R and mTOR are direct targets of both miR-99a and miR-497 **(A)** Putative binding sequences of IGF1R and mTOR with complementary sites for the seed regions of miR-99a and miR-497, as shown. **(B)** The analysis of the relative luciferase activities of IGF1R-WT and IGF1R-Mut in the Hep3B cells. **(C)** The analysis of the relative luciferase activities of mTOR-WT and mTOR-Mut in the Hep3B cells. NC: negative control microRNA. *P<0.05, **P<0.005, ***P<0.001, compared to NC.

To confirm the functional interaction between IGF1R/mTOR and miR-99a/miR-497, we performed luciferase experiment. In Figure 1A, 3′-UTR of IGF1R and mTOR contained a conserved putative target site for both miR-497 and miR-99a, respectively. Therefore, the 3′-UTR of human IGF1R or mTOR was amplified and inserted into down-stream of the luciferase gene in the psiCHECK-2 Vector. As shown in Figure 1B, 1C, miR-497 and miR-99a mimics repressed the luciferase activity, respectively. Mutation of miR-497 or miR-99a binding site from the target mRNA 3′-UTR largely abolished the effects of miR-497 and miR-99a mimics.

### MiR-497 and miR-99a were down-regulated in HCC tissue samples and cell lines

**Figure 2:**
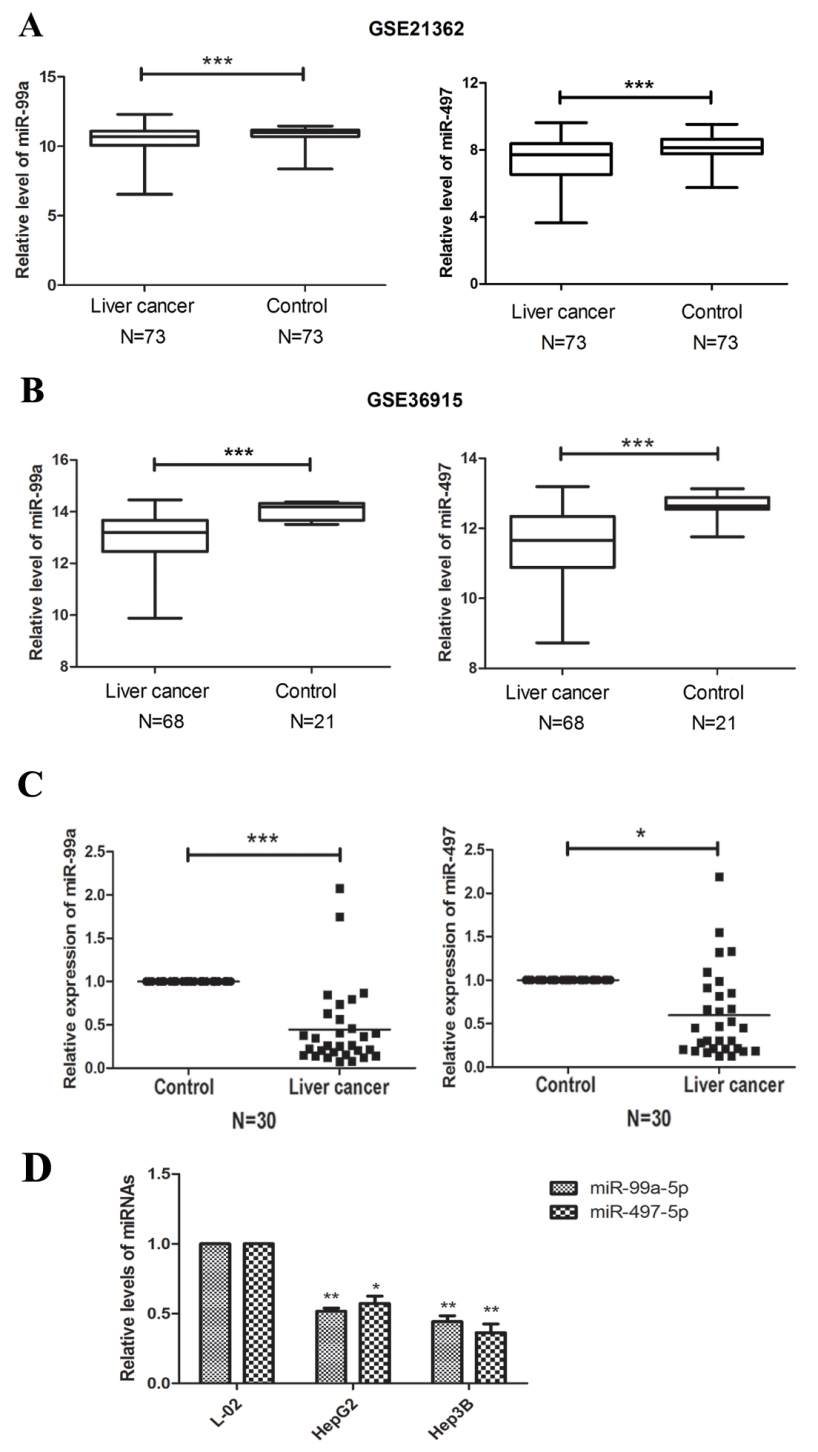
miR-99a and MiR-497 were down-regulated in HCC tissues and cell lines **(A, B)** Expression profiling of miRNAs showing that miR-99a and miR-497 were down-regulated in HCC tissues compared with non-cancerous liver tissues. The miRNA microarray data were obtained from NCBI, GEO database (Accession No: GSE21362 and GSE36915). The miRNA expression of HCC patients were illustrated by box plots. The median expression was indicated by horizontal line. **(C)** The qRT-PCR analysis for 30 paired HCC tissues and non-cancerous liver tissues. Both miR-99a and miR-497 were significantly down-regulated in HCC tissues compared to non-tumor tissues. **(D)** The qRT-PCR analysis of miR-99a and miR-497 for HCC cell lines (HepG2 and Hep3B) and liver normal cell lines (L-02). GAPDH was used as control. *P<0.05, **P<0.005, ***P<0.001, compared to control or L-02.

In GSE21362 and GSE36915, miR-497 and miR-99a were found to be significantly down-regulated in HCC tissues, compared with non-cancerous liver tissue (n=146 and 89, respectively; P<0.001; Figure 2A, 2B). Aberrant expression of miR-497 and miR-99a were further validated by qRT-PCR in 30 paired HCC and peri-tumoral tissue samples (Figure 2C). We also examined the expression of miR-497 and miR-99a in two HCC cell lines (HepG2 and Hep3B) and in one normal liver cell line L-02. The expression level of miR-497 and miR-99a in the two HCC cell lines was significantly lower than that in the L-02 cells (Figure 2D). These results indicated that expression of miR-497 and miR-99a were significantly decreased in HCC tumor tissues and HCC cell lines.

### IGF1R and mTOR were highly expressed and inversely correlated with the expression of miR-497 and miR-99a

**Figure 3:**
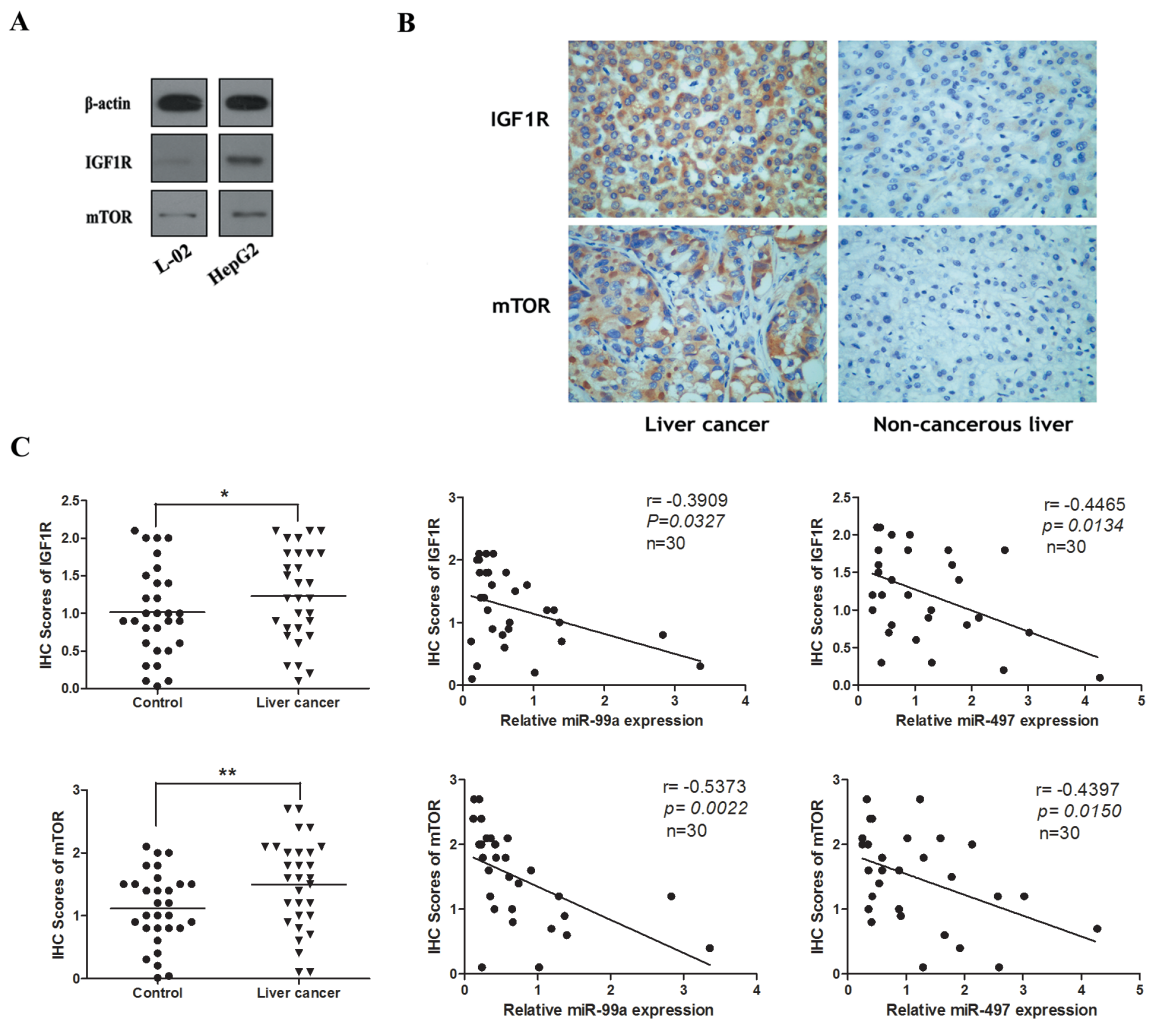
The expression of IGF1R and mTOR in HCC cell lines and tissues were inversely correlated with the levels of miR-99a and miR-497 **(A)** The protein levels of IGF1R and mTOR in HepG2 cell line were higher than them in liver normal cell lines (L-02). **(B)** Representative immunohistochemical staining for IGF1R and mTOR from HCC tissues and non-tumourous liver tissues. Upper: IGF1R immunostaining; Lower: mTOR immunostaining (×200). **(C)** The immunohistochemistry(IHC) scores of IGF1R and mTOR in HCC tissues were significantly higher than them of matched non-cancerous liver tissues. *P < 0.05, **P<0.005, compared to control. And correlation analyses were performed between IHC scores of IGF1R/mTOR and the mRNA levels of miR-99a/miR-497 expression in HCC tissues (n = 30).

The expression levels of IGF1R and mTOR were determined in human tissues and cell lines using Western blot and immunohistochemistry. As illustrated in Figure 3A, 3B, dramatical upregulation of IGF1R and mTOR proteins were observed in HCC tissues and cell lines as compared with peri-tumoral tissues and normal liver cell line L-02. The immunostaining of IGF1R and mTOR of individual cases were scored. Moreover, we analyzed the protein expression of IGF1R and mTOR by immunohistochemical staining in patients with HCC. As shown in Figure 3C, the immunostaining intensity of IGF1R and mTOR in HCC tissues were obviously higher than that of adjacent non-tumourous tissues. Next we explored the potential relationship between IGF1R/mTOR and miR-497/miR-99a, and found that IGF1R/mTOR and miR-497/miR-99a were significantly negatively correlated (Figure 3C). These data indicates that up-regulated IGF1R and mTOR were inversely correlated with the decreased expression of miR-497 and miR-99a in HCC.

### MiR-497 and miR-99a inhibited HCC cell growth *in vitro*, which were reversed by up-regulating IGF1R and mTOR

**Figure 4:**
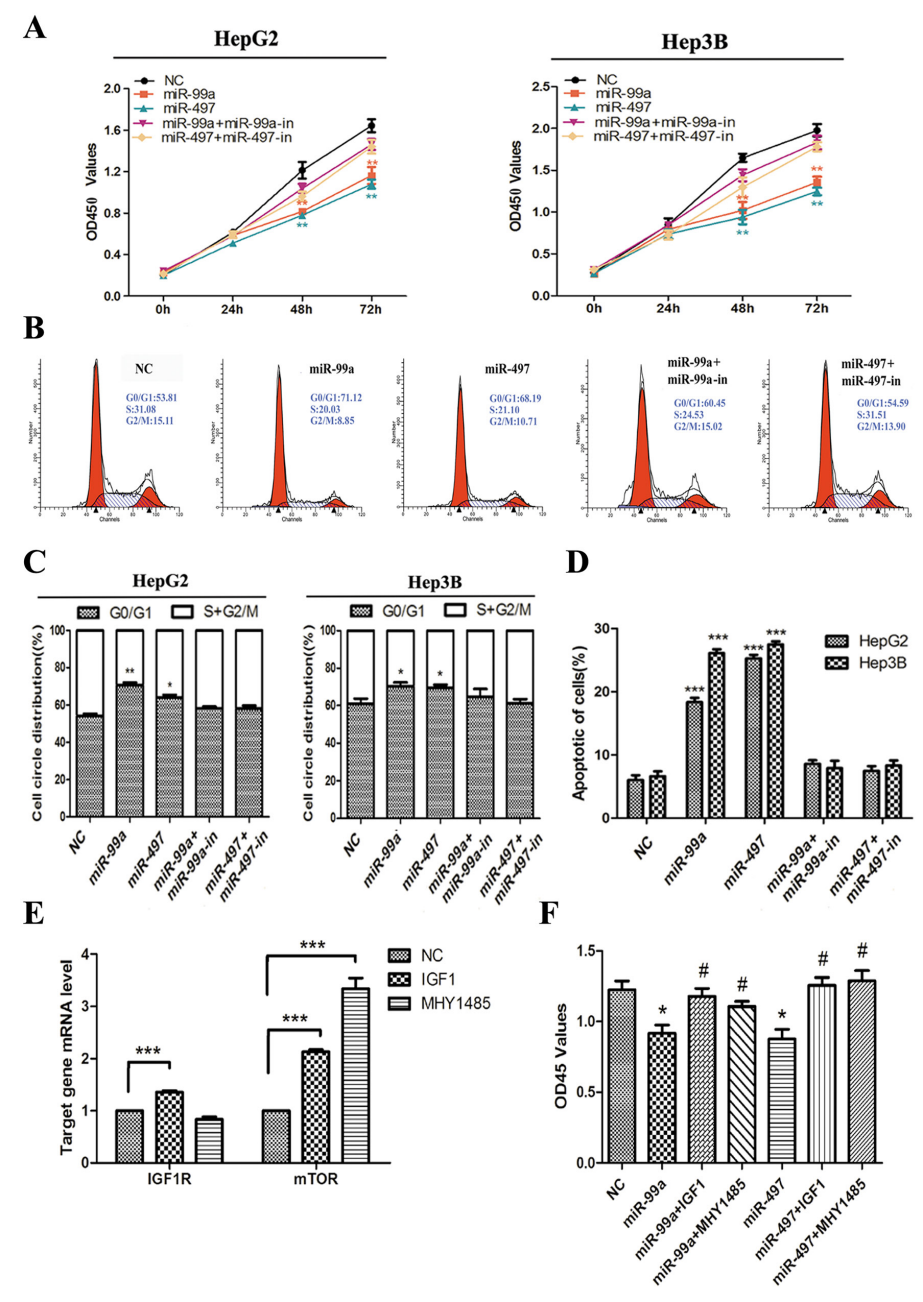
Up-regulation of miR-99a and miR-497 inhibited liver cancer cell proliferation, delayed cell cycle progression, and promoted apoptosis in liver cancer cell, which were reversed by up-regulating IGF1R and mTOR **(A)** Ectopic expression of miR-99a and miR-497 suppressed HegG2 and Hep3B cell lines proliferation at 48h and 72h. And these anti-growth effects would be attenuated after co-transfection with antisense miR-99a and miR-497 (miR-99a-in and miR-497-in). **(B)** After transfection of miR-99a and miR-497 mimics or co-transfection with miR-99a-in and miR-497-in to HCC cells, the DNA content of PI-stained cells were analyzed by flow-cytometry. Cell-cycle distribution of G0/G1 phase presented here was in HepG2 cells. **(C)** The stained cell number ratios of HepG2 and Hep3B cell lines are presented in bar graph. **(D)** Evaluation of apoptosis by annexin V-PITC (AV) and propidium iodide (PI) staining and analysis by flow-cytometry in HepG2 and Hep3B cells after transfection. The apoptotic cell number are counted in bar graph. **(E)** Validation of drug-mediated IGF1R and mTOR activating in HepG2 cells. The relative IFG1R and mTOR mRNA levels, as determined by real-time qPCR, are expressed as fold changes after normalization to the internal control (β-actin). **(F)** Cell growth was monitored in HepG2 cells at 48h using the CCK-8 assay. NC: negative control microRNA. *P<0.05, **P<0.005, ***P<0.001, compared to NC. ^#^P<0.05, compared to transfection with miR-99a or miR-497.

Concerning the significant down-regulation of miR-497 and miR-99a in HCC tissues and cell lines (Figure 2), their functional role in HCC carcinogenesis was investigated. By ectopical expression of miR mimics and/or inhibitors as well as corresponding non-targeting control miRNAs in HCC cell lines, the expression of miR-497 or miR-99a in tumor cells were efficiently manipulated (Supplementary Figure 2). As illustrated in Figure 4A, up-regulation of miR-497 or miR-99a significantly decreased the growth rate of HepG2 and Hep3B cells. This inhibition is mediated by retaining more cells in G0/G1 phase from entering into S phase of the cell cycle and increasing apoptosis of the tumor cells (Figure 4B-4D). But this inhibitory effect of miR-497 and miR-99a on cell proliferation was entirely abolished by co-transfecting cells with miR inhibitors (Figure 4A-4D) and completely reversed by application of IGF1 (IGF1R ligand) or MHY1485 (mTOR agonist) (Figure 4F). Significant down-regulation of IGF1R and/or mTOR mRNA were observed in IGF1 or MHY1485 treated group (Figure 4E). Therefore, we thought the inhibitory effects of miR-497/miR-99a on HCC cell growth is through repressing IGF1R/mTOR signalling.

### MiR-497 and miR-99a suppressed HCC tumor invasiveness

**Figure 5:**
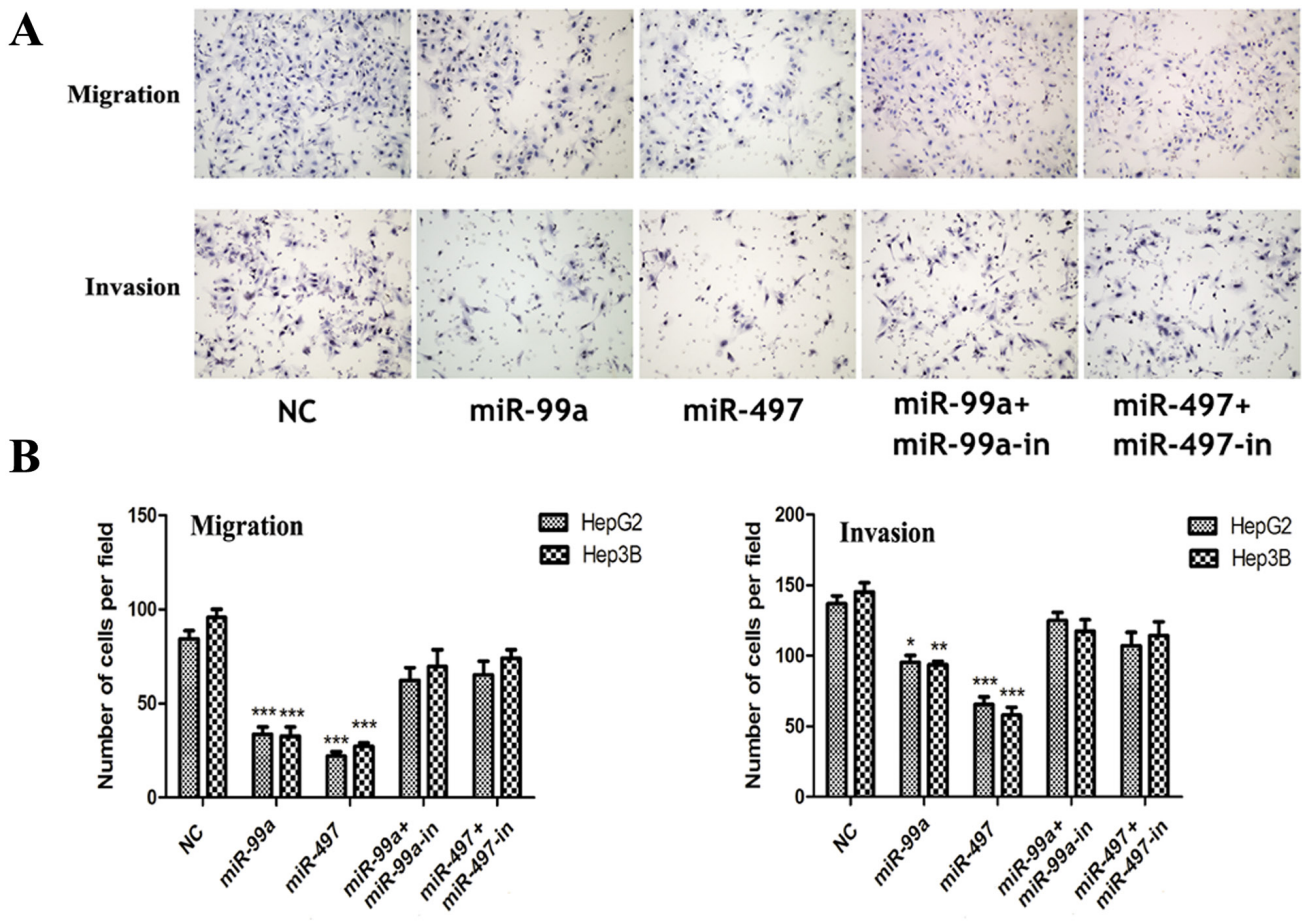
Up-regulation of miR-99a and miR-497 inhibited liver cancer cell migration and invasion **(A)** Up-expression of miR-99a and miR-497 could significantly inhibit HepG2 and Hep3B cells migration and invasion whereas these effects were reversed when co-transfection with miRNAs inhibitors (presented here was in HepG2 cells). **(B)** The relative cells of each group were shown in bar graphs. NC: negative control microRNA. *P<0.05, **P<0.005, ***P<0.001, compared to NC.

To investigate the function of these two miRNAs on tumor invasiveness, transwell assays were performed. We found that exogenous expression of miR-497 or miR-99a in HCC cells resulted in markedly suppressed migration and invasion in HepG2 and Hep3B cells through membrane or matri-gel coated membrane (Figure 5A, 5B). Such inhibitory effect on cell migration and invasion were totally abolished in miRNA mimics and miRNA inhibitor coexpression groups (Figure 5A, 5B).

Collectively, our results suggested that miR-497 or miR-99a inhibited HCC cell growth and invasion capacities and functioned as tumor suppressors.

### Synergistic effects of miR-497 and miR-99a on inhibiting HCC cell proliferation by co-targeting IGF1R and mTOR

**Figure 6:**
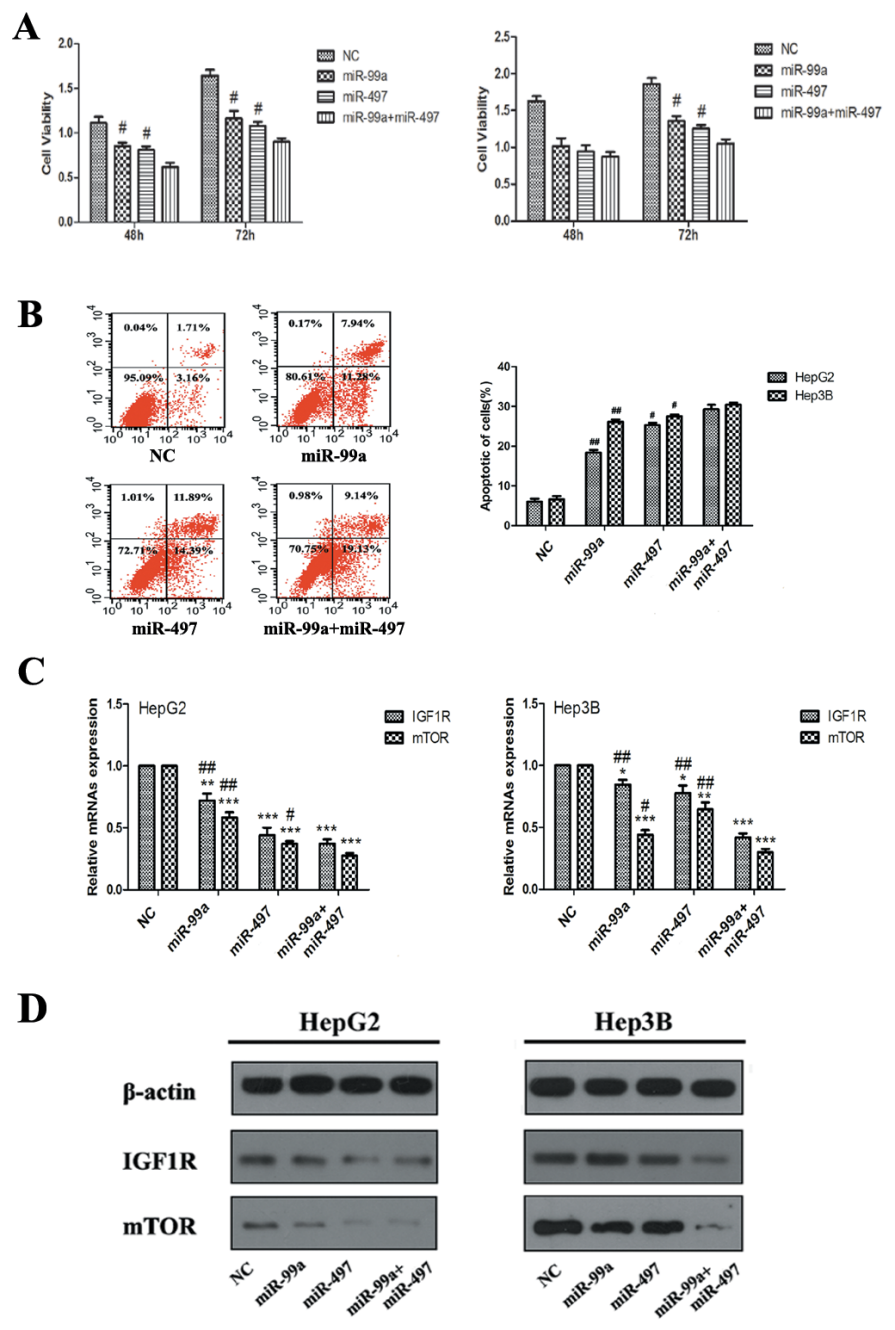
Synergistic effects of miR-497 and miR-99a on retarding HCC cell proliferation, which related with synergistic co-targeting on IGF1R and mTOR **(A)** Co-expression of miR-99a and miR-497 exerted stronger suppression on HegG2 and Hep3B cell proliferation than that of miR-497 or miR-99a alone at 48h and/or 72h. **(B)** Evaluation of apoptosis by flow-cytometry in HepG2 and Hep3B cells transfected with of miR-99a and/or miR-497 (apoptotic cell distribution phase presented here was in HepG2 cells). The apoptotic cell number are counted in bar graph. **(C)** The levels of IGF1R and mTOR mRNAs analyzed by qRT-PCR, GAPDH was used as control. **(D)** The protein levels of IGF1R and mTOR were detected by Western blot assay,β-actin was used as control. The results were reproducible in three independent experiments. NC: negative control microRNA. *P<0.05, **P<0.005, ***P<0.001, compared to NC. ^#^P<0.05, ^##^P<0.005, compared to co-transfection with miR-99a and miR-497.

As both IGF1R and mTOR are targets of miR-497 and miR-99a, we examined whether these two miRNAs exert much stronger effects on HCC cell proliferation and apoptosis than individuals. As showed in Figure 6A, HepG2 and Hep3B cells co-expressing miR-497 and miR-99a exhibited a more significant reduction in cell viability than the cells transfected with miR-497 or miR-99a alone (P<0.05; HepG2 cell at 48h and 72h, Hep3B cell at 72h), which is found to be statistically synergistic. Additionally, we found that the percentage of apoptotic cells in co-expressing group was much higher too (Figure 6B).

Since miR-497 and miR-99a have synergistic effects on HCC cell proliferation, further examination of whether these two miRNAs have synergistic effects on IGF1R and mTOR need to be carried out. As demonstrated in Figure 6C, the mRNA levels of IGF1R and mTOR were significantly decreased in miR-497 or miR-99a over-expressed cells, and the expressions were further down-regulated in the cells co-expressing both two miRNAs. Western blot analysis further revealed the down-regulated expression of IGF1R and mTOR at protein level, and a synergistic down-regulation of IGF1R and mTOR was also observed (Figure 6D). Taken together, our results suggest that miR-497 and/or miR-99a can work as tumor suppressor in combination synergistically inhibit tumor growth via co-targeting IGF1R and mTOR.

### miR-497 and/or miR-99a retarded HCC development *in vivo*

**Figure 7:**
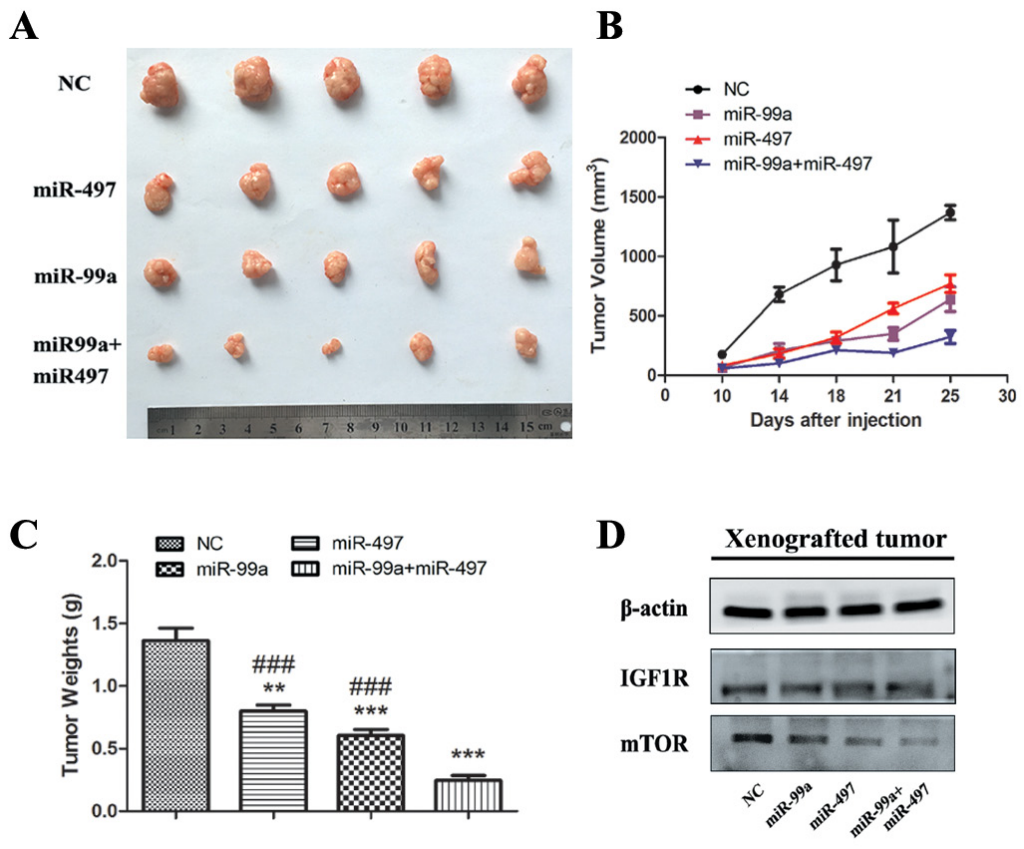
MiR-99a and miR-497 repressed the growth of HepG2-engrafted tumors and decreased the expression of IGF1R and mTOR in vivo **(A)** Engrafted tumors from scrambled and miR-mimics-treated mice groups were dissected and photographed at the day 25 after transplantation. **(B)** Tumor volumes at the indicated days during the experiment for the groups. **(C)** Tumor weights averages between scrambled and miR-mimics-treated mice groups at the day 25 after engraftment. **(D)** Western blotting analyses of the protein levels of IGF1R and mTOR in HepG2-engrafted tumors. β-actin was used as control. The results were reproducible in three independent experiments. NC: negative control microRNA. **P<0.005, ***P<0.001, compared to NC. #P<0.05, ##P<0.005, ###P<0.001, compared to co-transfection with miR-99a and miR-497.

To further confirm the tumor suppressive function of miR-497 and miR-99a, HepG2 cells were engrafted into nude mice. As shown in Figure 7A-7C, at 25 days, the tumor volume and weight were markedly decreased in miR-99a or miR-497 over-expressed tumors compared to the control as IGF1R and mTOR were down-regulated in the xenograft tumors (Figure 7D). Such inhibition on the tumor growth was synergistically strengthened in miR-497 and miR-99a co-expressing group via synergistically suppressing IGF1R and mTOR, which is consistent with the observation in the *in vitro* assay. Thus, miR-497 and miR-99a can synergistically functions as tumor suppressors *in vivo*.

## DISCUSSION

MiRNAs have been demonstrated to negatively regulate target genes in a sequence-specific manner, and are key molecular regulators in a wide variety of oncogenic processes, functioning as potential diagnostic and prognostic biomarkers [20]. Therefore, elucidating the underlying mechanism of miRNAs in HCC development may provide valuable diagnostic and therapeutic strategies for malignancy. Our study showed that miR-497 and miR-99a, the direct targets of IGF1R and mTOR, were significantly down-regulated in HCC tissues and cell lines, and they repressed certain aspects of tumorigenesis by targeting IGF1R and mTOR. While co-expression of these two miRNAs could have synergistic effect on HCC cell growth, predominantly through their co-operative effects on the down-regulation of IGF1R and mTOR.

According to the NCBI, GEO database, we generalized the repressive status of miR-497 and miR-99a in HCC by recapitulating their expressions from the large cohorts of HCC patients (Figure 2A, 2B). Obviously miR-497 and miR-99a appeared to be suppressed in HCC compared with non-cancerous surrounding tissues. This prompted us to investigate whether these two miRNAs function as anti-tumor agents. From our data, it was indicated that miR-497 and miR-99a were significantly down-regulated in patients with HCC and HCC cell lines. The expression of miRNAs in cancer was deregulated by several mechanisms, such as epigenetic mechanisms (DNA methylation and histone modification), chromosome deficiency or duplication, abnormal transcription factors, and disordered microRNA maturation [21–23]. In our study, miR-497 and miR-99a are located on chromosomes 17p13.1 and 21q, respectively. The loss or deletion of chromosome 17p13.1 has been reported in various types of cancer, including HCC, which suggested that the down-regulation of miRNA-497 in HCC probably arises from genomic DNA loss or deletion [24]. In addition, DNA methylation was also related to the down-regulation of miRNA-497 in tumors, such as ovarian and breast cancer [25, 26]. The gene encoding miR-99a was found residing within an intron of C21orf34, which was reported harboring a putative tumor suppressor gene in HCC [21]. Inhibited DNA repair proteins which were essential for the maintenance of genomic integrity would subsequently suppressing the expression of miR-99a [27]. These alterations may help us to understand the down-regulation of miR-497 and miR-99a and thereby contribute to the hepatocellular malignant transformation. Further studies on the suppression of miR-497 and miR-99a should be elucidated in the future.

As reported previously, miR-497 retards lung cancer cell growth or invasion by targeting CCNE1 [28], VEGFA [29], whereas miR-497 does so by targeting SSRP1 [30] in HCC. Our study extends the results of others by showing that miR-497 targets both IGF1R and mTOR in HCC. It is known that IGF1R and mTOR are crucial factors for the regulation of cell survival and cell proliferation [8–10]. In this study, luciferase reporter assay indicated that miR497 directly bind to the 3’ UTR of IGF1R/mTOR, and down-regulated mRNA expression of IGF1R/mTOR. The interaction between miRNA-497 and IGF1R/mTOR were further collaborated by the inversely correlated expressions of miR-497 and IGF1R/mTOR in HCC tumor tissue and cell lines. Up-regulating miR-497 in HepG2 and Hep3B cell lines and xenograft models resulted in the down-regulation of IGF1R and mTOR and inhibition of tumor growth and cell invasion. Another microRNA, miR-99a, is involved in some important signaling pathways, such as AKT [31], FGFR [32], and modulate TGF-βpathway induced epithelial to mesenchymal transformation in breast [33]. There is already evidence that mTOR and its upstream activator, IGF1R, are the direct targets of miR-99a in lung cancer, esophageal squamous cell carcinoma, acute lymphoblastic leukaemia as well as HCC [21, 34–36]. In this study, we demonstrated that ectopic expression of miR-99a in HepG2 and Hep3B cell lines and tumor models could suppress cell proliferation, apoptosis, invasive, metastasis and tumor growth by targeting IGF1R and mTOR, which were consistent with the previous observation [21]. These and our results, together with the high possibility that more miR-497 or miR-99a common targets will be discovered soon, suggest that these two miRNAs share overlapping functions, regulate important signaling pathway-related mRNAs (including IGF1R and mTOR), and thus function as tumor suppressors.

As these two miRNAs were both down-regulated in HCC and inversely correlated with same targets, we speculated that miR-497 and miR-99a might be co-operative on the growth of HCC cells by co-targeting these two genes. To validate this hypothesis, we co-transfected miR-497 and miR-99a into HepG2 and Hep3B cell lines, and found that these two miRNAs act synergistically on retarding HCC cell proliferation via co-targeting IGF1R and mTOR. The same results were also found on xenograft models. To the best of our knowledge, this is the first study to demonstrate the co-operative effect of miR-497 and miR-99a on inhibiting HCC cell growth. Recent studies have focused on the effects of miRNA clusters axis on cancer, and have demonstrated that miRNAs encoded in the same miRNA cluster function synergistically [37–40]. Besides, though some miRNAs belong to different clusters, they have the same seed sequence or proximal seed sequences on targets and can also act synergistically on tumorigenesis or immune reaction [28, 41]. While up to now, there are only few reports of miRNAs which contain completely unrelated seed sequence act in a symergistic manner [42–45]. Bandi et al. reported that miR-34a and miR-15a/16 potentiate their impact on G1-S progression in non-small cell lung cancer cells [42]. And they thought the synergistic effect can rather be explained by the fact that in concerted action both miRNAs are able to down-regulate more targets than each miRNA alone. However, they drew this conclusion only by detecting the mRNA levels of target genes in single or dual miRNA group, and found there was no significant differences. The post-transcriptional regulation of the target gene by miRNA was not included in consideration. In this study, we demonstrated that miR-497 and miR-99a from different miRNA clusters and having different seed sequences, act synergistically on tumor cell proliferation and growth. Real-time PCR and western blot assay further showed that miR-497 and miR-99a suppressed the expression of IGF1R and mTOR in a synergistic manner. Apparently the synergistic inhibition of miR-497 and miR-99a on tumor growth could be interpreted by their synergistical inhibition on expression of IGF1R and mTOR. The binding sites proximity of these two miRNAs can largely influence the protein translation [46]. Besides, It is known that miRNA has the capacity to simultaneously regulate many different targets across multiple pathways. Both miR-497 and miR-99a modulate other oncogenes that may mediate their tumor suppressor function. P70S6K (miR-497) [25], and FGF family (including FGFR1 and FGFR3, targeted by miR-497 and miR-99a, respectively) [47, 32] are involved in the downstream genes of IFG1R/mTOR pathway. TSC1 (putative target of miR-497 identified by TargetScan) can regulate the activity of mTOR. Therefore, the combination of miR-497 and miR-99a could be superior to each individual miRNA alone in retarding HCC cell growth. But the relationship between miR-497 and miR-99a is complex and their co-operative effect on HCC growth requires further research.

In conclusion, the major finding of our study is that miR-497 and miR-99a synergistically target IGF1R and mTOR, thereby impeding the HCC tumor growth. These results promote a concept in which not one single miRNA, but rather a network of miRNAs with shared and individual mRNA targets participates in the hepatocarcinogenesis. Simultaneous targeting of IGF1R and mTOR by co-transfecting two miRNAs yields promising results for eradicating HCC cells, which could be a new direction for liver cancer treatment.

## MATERIALS AND METHODS

### Patients and samples

The study was approved by the Institutional Ethics Committee. Tissue samples were taken from central part of the tumors and adjacent non-cancerous liver tissues of HCC patients (n=30) hospitalized at Tongji Hospital, Tongji Medical College, Huazhong University of Science and Technology, from 2010 to 2013. All samples were formalin-fixed and paraffin-embedded (FFPE), and the slides were reviewed by two pathologists independently and diagnosed as hepatocellular carcinoma. None of these patients had received local or systemic anticancer treatment before surgery. The information of clinical characteristics of all 30 patients, including 6 in clinical stage I, 9 in clinical stage II, 12 clinical stage III, 3 clinical stage VI. The study was approved by the Ethics Committee of Tongji Hospital, Tongji Medical College, Huazhong University of Science and Technology, Wuhan, China. All experiments were performed in accordance with relevant guidelines and regulations. Written informed consent was obtained from the patient.

### Cell culture

HCC cell lines, Hep2G, Hep3B, purchased from the American Type CultureCollection (ATCC, Manassas, VA, USA), were maintained in Dulbecco's modified Eagle's medium (Hyclone, Logan, UT, USA) supplemented with 10% fetal bovine serum (Hyclone, ThermoFisher Scientific, Mordialloc, Victoria, Australia), within a humidified atmosphere containing 5% CO2 at 37°C. Normal liver epithelial cell L-02 was purchased from the Chinese Academy of Sciences Committee Type Culture Collection cell bank and was cultured under the conditions stated by the manufacturer.

### Luciferase reporter assay

The 3’-UTR of IGF1R and mTOR containing the putative binding sites of miR-99a or miR-497 and their corresponding mutated sequences were amplified and verified by DNA sequencing. These gene fragments were then subcloned down-stream from the Renilla luciferase gene in the psiCHECK2(Promega) to generate the wild-type IGF1R and mTOR plasmids (IFG1R 3’-UTR WT, mTOR 3’-UTR WT), the mutant IGF1R and mTOR plasmids(IFG1R 3’-UTR Mut, mTOR 3’-UTR Mut). For luciferase assays, Hep3B cells were co-transfected with miR-99a or miR-497 mimics and corresponding plasmid in 24-well plates using Lipofectamine 3000 (Invitrogen). The cells were collected 48 h after transfection, and the luciferase activity was measured with the Dual-Luciferase Reporter Assay System (Promega).

### RNA extraction and real-time quantitative PCR(qRT-PCR)

The TRIzol reagent (Invitrogen) was used to extract total RNA from cultured cells according to the manufacturer's protocol. Complementary DNAs were synthesized and Real-time PCR was performed on a StepOnePlus™ Real-Time PCR Systems (Applied Biosystems international, Inc. Delaware) using SYBR Green (Qiagen, Shanghai, China). GAPDH or β-actin were used as an internal controls. The following primers were synthesized and used in this study: ***IGF1R*** (F 5’-CGATGTGTGAGAAGACCACCA-3’; R: 5’-ACATTTCTGGCAGCGGTTT-3’); ***mTOR*** (F 5’-CTGCGACTCAAATGTGTGCAG-3’; R: 5’-GAACAATAGGGTGAATGATCCGGG-3’). Total miRNA from cultured cells and FFPE HCC tissue samples was extracted using the RecoverAll™ Total Nucleic Acid Isolation Kit (Ambion, TX, USA) according to the manufacturer's manual. The expression level of miR-99a and miR-497 were performed on an ABI 7900 system (Applied Biosystems). The expression level of U6 snRNA was used as an internal control for normalization. The following miR-specific primers were synthesized and used in this study: ***miR-99a*** (F: 5’-AGAGCAACCCGTAGATCCGA-3’; R:5’-CAGTG CAGGGTCCGAGGT-3’); ***miR-497*** (F: 5’-CCTTCAG CAGCACACTGTGG-3’; R:5’-CAGTGCAGGGTCCGA GGTAT-3’); ***U6*** (F: 5’-GCGCGTCGTGAAGCGTTC-3’; R: 5’-GTGCAGGGTCCGAGGT-3’).

### Oligonucleotides and transfection

MiR-99a and miR-497 mimics/inhibitors and negative control molecules were purchased from (Ribo, guangzhou, China). Cell transfection was performed using Lipofectamine (Life Technologies) until a final concentration of 20 nM. Medium was changed after 6 h. After transfected and cultured for 48 h, cells were collected for qRT-PCR and Western blot analyses.

### Cell proliferation assay

Cell proliferation was assessed using Cell Counting Kit-8(CCK-8) assay according to the manufacturer's protocol. Cells were cultured in 10% CCK-8 (Dojindo, Kumamoto, Japan) diluted in normal culture media at 37°C. When visual color conversion appeared, quantification was carried out on an automated plate reader (Bio-Rad SmartSpec Plus, USA) at a wavelength of 450 nm.

### Flow cytometry analysis

Cells in a culture dish were harvested by trypsinization, washed in ice-cold PBS and fixed in pre-chilled 70% ethanol at -20°C overnight. For measurement of DNA content, cells were stained with propidium iodide(PI) staining buffer (PBS, 50μg/ml PI, 0.1mg/ml DNase-free RNase), and incubated at room temperature in the dark for 30 min. DNA content was examined by flow cytometry (Becton Dickinson, North Ride, NSW, Australia) and analyzed using ModFit LT software (version 2; Verity Software House, Topsham, ME).

### Motility and invasion assay

For *in vitro* cell motility and invasion assay, Transwell plates and cell culture inserts (BD Biosciences, San Jose, CA) were used. For the coating of invasion assay, Matrigel (Corning Inc, Corning, NY, USA) was diluted to 0.3 mg/ml concentration and coated onto upper compartment of cell culture insert. After transfection of miR-99a and miR-497, HepG2 and Hep3B cells (5 × 10^4^ cell/well for motility assay, 1 × 10^5^ cell/well for invasion assay) were transferred on the top of the cell culture insert with DMEM/F12(Sigma-Aldrich, MO, USA) and 5% fetal bovine serum. After 4 h (motility) or 12 h (invasion) of incubation at 37 °C, migrated or invaded cells were fixed with 1% paraformaldehyde, stained with hematoxylin and photographed with Axiovert 200 inverted microscope at ×200 magnification. The cell number was counted in three random fields of view.

### Western blotting

Cellular and tissue proteins were prepared in extraction-buffer (Thermo Fisher Scientific Pierce, Rockford, USA). Equal quantities of protein were electrophoresed through a 12% SDS–PAGE gel and transferred to a nitrocellulose membrane. The membranes were incubated with anti-IGF1R (1:200; bs-5448R Bioss-bio, Beijing, China), anti-mTOR (1:200; bs-3559R Bioss-bio), anti-β-catenin (1:200; bs-1165R Bioss-bio). The membranes were stripped and re-blotted with HRP-conjugated secondary antibodies and visualized by ECL (Thermo Pierce, Cramlington, UK) as loading control. Images were captured on Kodak Image Station and the density of the band immunoreactive for the protein was normalized to the intensity of β-actin band.

### Immunohistochemistry

Immunohistochemistry(IHC) stainings were carried out on 4 μm FFPE sections with antibodies as described below. Briefly, the slides were de-paraffinized, re-hydrated, and dripped in 3% H_2_O_2_ for 10 minutes, then incubated with IGF1R and mTOR antibodies at 4°C for overnight. After washing with PBS, the specimens were then incubated with Polymer Helper for 20 minutes, and followed by polyperoxidase–anti-human IgG for 30 minutes at room temperature. Development of the slides was carried out using EnVision Detection Kit (Maxin, China). Counterstaining was performed with hematoxylin, and the substitution of PBS for primary antibody was used as negative control. Finally the slides were examined by two pathologists blindly and independently. Staining intensity was scored as follows: no staining received a score of 0, weak staining received a score of 1, moderate staining received a score of 2 and strong staining received a score of 3. Tumor cells were randomly selected and counted based on the percentage of positively stained cells (0–100%). The final IHC score was calculated by multiplying the intensity score with the percentage of positive cells. Maximum = 3.

### Tumorigenicity assay *in vivo*

All experimental procedures were carried out according to institutional guidelines. The HepG2 cells were propagated and 5×10^6^ cells were injected subcutaneously into the posterior flanks of BALB/c-nu mice (5-6 weeks). 7 days later, the mice were then intratumorally injected with antagomir control or antagomir-497 and/or antagomir-99a and 3 times per week for 3 weeks. Tumors were examined twice weekly; length and width were obtained with vernier caliper and tumor volumes were calculated using the equation volume (mm^3^) = (length × width ^2^)/2. On the 25th day after injection, mice were killed. Tumors were collected and weighted after necropsy.

### Statistical analysis

All data were expressed as the mean ± SD. The Student's t-test was used to evaluate the statistical significance of differences between two groups of data in all pertinent experiments. Paired or unpaired t-test was used if the samples were matched or not. The Pearson Correlation was used to determine the statistical significance of miR-497/miR-99a expression and immunohistochemistry(IHC) scores of IGF1R and mTOR. Two-way ANOVA was used to assess the synergistic effect of miR-497 and miR-99a. Statistical analysis was performed using IBM SPSS Statistics V17.0 (IBM Corporation). P < 0.05 was thought to be significantly different for two groups of data.

## SUPPLEMENTARY MATERIALS FIGURES AND TABLES




