# Supplementary material for: Co-targeting of IGF1R/mTOR pathway by miR-497 and miR-99a impairs hepatocellular carcinoma development
Source: Oncotarget. 2017 May 24;8(29):47984–97. doi: 10.18632/oncotarget.18207 (PMC5564620; doi:10.18632/oncotarget.18207)
Supplement: Supplementary file 1 [file oncotarget-08-47984-s001.pdf]

Co-targeting of IGF1R/mTOR pathway by miR-497 and miR-99a impairs hepatocellular carcinoma development

SUPPLEMENTARY MATERIALS

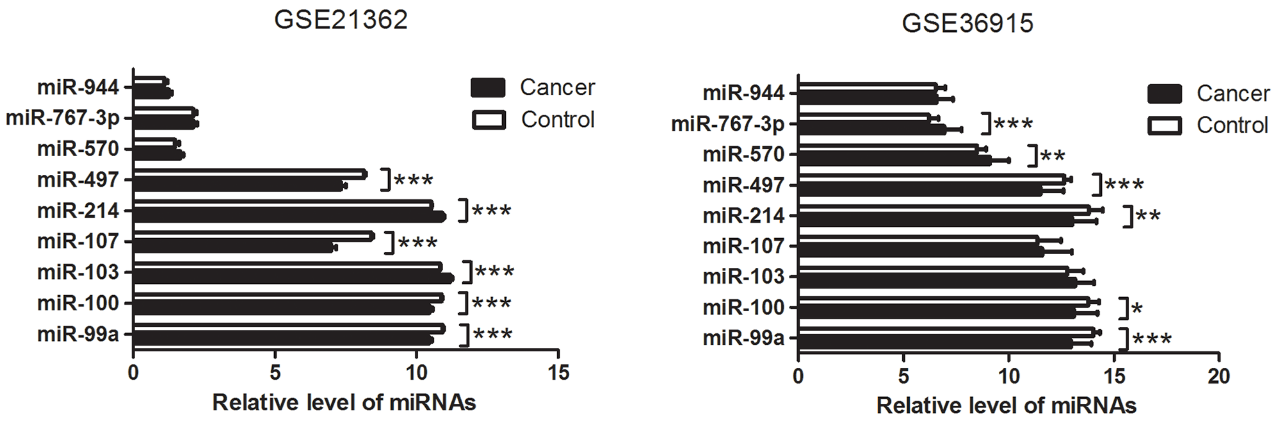

Supplementary Figure 1: Common miRNAs of both IFG1R and mTOR expressed in GSE21362 and 36915. N=146 and 89, respectively; *P<0.05, **P<0.005, ***P<0.001, compared to control.

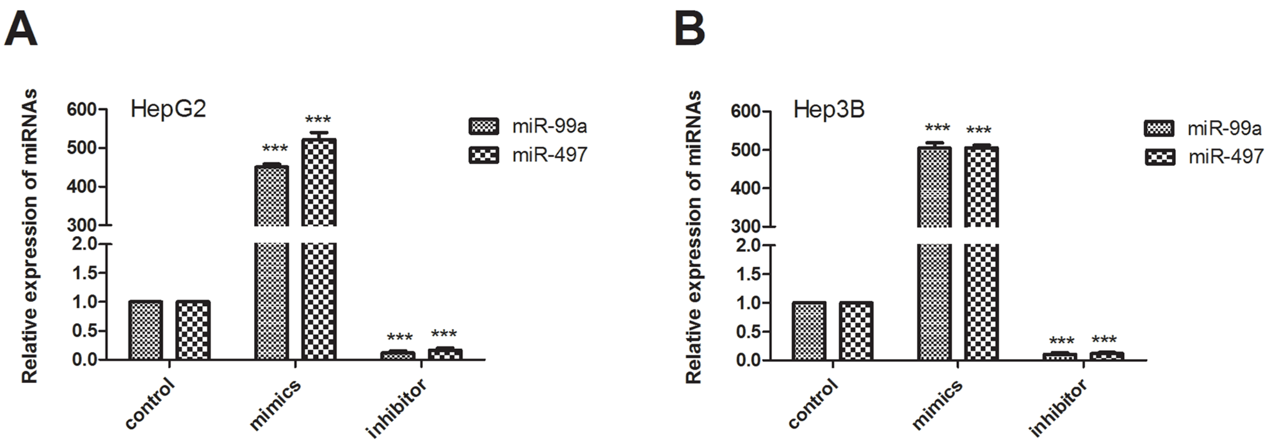

Supplementary Figure 2: The expression level of miRNAs in HepG2 and Hep3B cells. The expressions of miR-99a and miR-497 in control group were both set to 1. miR-99a and miR-497 mimics enhance the corresponding miRNA expression, while miR-99a and miR-497 inhibitors can repress the expression in **(A)** HepG2 and **(B)** Hep3B cells. ***P<0.001, compared to control.

Supplementary Table 1: Predicted target miRNAs and common miRNAs of IGF1R and mTOR in 10 poplular miRNA databases.

See Supplementary File 1
