# Supplementary material for: Co-targeting of IGF1R/mTOR pathway by miR-497 and miR-99a impairs hepatocellular carcinoma development
Source: Oncotarget. 2017 May 24;8(29):47984–97. doi: 10.18632/oncotarget.18207 (PMC5564620; doi:10.18632/oncotarget.18207)
Supplement: Supplementary file 2 [file oncotarget-08-47984-s002.doc]

**Supplementary Table 1:** Predicted target miRNAs and common miRNAs of IGF1R and mTOR in 10 poplular miRNA databases

| **Target gene** | **miRNAs** | **Target gene** | **miRNAs** | **Common miRNAs** |
| --- | --- | --- | --- | --- |
| IGF1R | hsa-mir-182 | mTOR | hsa-mir-99a | hsa-mir-99a |
|  | hsa-let-7c |  | hsa-miR-100 | hsa-miR-100 |
|  | hsa-miR-22 |  | hsa-miR-96 | hsa-miR-103 |
|  | hsa-let-7b |  | hsa-miR-511 | hsa-miR-107 |
|  | hsa-mir-99a |  | hsa-miR-581 | hsa-miR-214 |
|  | hsa-miR-96 |  | hsa-miR-646 | hsa-miR-497 |
|  | hsa-let-7d |  | hsa-miR-99b | hsa-miR-570 |
|  | hsa-miR-185 |  | hsa-miR-421 | hsa-miR-767-3p |
|  | hsa-let-7g |  | hsa-miR-767-3p | hsa-miR-944 |
|  | hsa-miR-646 |  | hsa-miR-515-5p |  |
|  | hsa-miR-302d |  | hsa-miR-338-5p |  |
|  | hsa-miR-153 |  | hsa-miR-7 |  |
|  | hsa-let-7i |  | hsa-miR-659 |  |
|  | hsa-let-7e |  | hsa-miR-101 |  |
|  | hsa-miR-597 |  | hsa-miR-496 |  |
|  | hsa-miR-619 |  | hsa-miR-660 |  |
|  | hsa-miR-302a |  | hsa-miR-199a-3p |  |
|  | hsa-miR-16 |  | hsa-miR-144 |  |
|  | hsa-miR-769-5p |  | hsa-miR-199b-3p |  |
|  | hsa-miR-15a |  | hsa-miR-485-5p |  |
|  | hsa-miR-187 |  | hsa-miR-578 |  |
|  | hsa-miR-217 |  | hsa-miR-214 |  |
|  | hsa-miR-223 |  | hsa-miR-579 |  |
|  | hsa-miR-202 |  | hsa-miR-1271 |  |
|  | hsa-miR-588 |  | hsa-miR-944 |  |
|  | hsa-miR-608 |  | hsa-miR-193a-5p |  |
|  | hsa-miR-195 |  | hsa-miR-323-5p |  |
|  | hsa-miR-661 |  | hsa-miR-103 |  |
|  | hsa-miR-133a |  | hsa-miR-520g |  |
|  | hsa-miR-766 |  | hsa-miR-370 |  |
|  | hsa-miR-892a |  | hsa-miR-33b |  |
|  | hsa-miR-302c |  | hsa-miR-298 |  |
|  | hsa-miR-10b |  | hsa-miR-107 |  |
|  | hsa-miR-152 |  | hsa-miR-582-5p |  |
|  | hsa-miR-184 |  | hsa-miR-450b-3p |  |
|  | hsa-miR-567 |  | hsa-miR-339-5p |  |
|  | hsa-miR-520a-5p |  | hsa-miR-545 |  |
|  | hsa-miR-599 |  | hsa-miR-604 |  |
|  | hsa-miR-644 |  | hsa-miR-101 |  |
|  | hsa-miR-98 |  | hsa-miR-889 |  |
|  | hsa-miR-662 |  | hsa-miR-1229 |  |
|  | hsa-miR-103 |  | hsa-miR-559 |  |
|  | hsa-miR-34b |  | hsa-miR-607 |  |
|  | hsa-miR-145 |  | hsa-miR-1244 |  |
|  | hsa-miR-452 |  | hsa-miR-424 |  |
|  | hsa-miR-153 |  | hsa-miR-497 |  |
|  | hsa-miR-570 |  | hsa-miR-135a |  |
|  | hsa-miR-497 |  | hsa-miR-570 |  |
|  | hsa-miR-623 |  | hsa-miR-375 |  |
|  | hsa-miR-552 |  | hsa-miR-627 |  |
|  | hsa-miR-206 |  | hsa-miR-936 |  |
|  | hsa-miR-765 |  | hsa-miR-129-3p |  |
|  | hsa-miR-509-5p |  |  |  |
|  | hsa-miR-885-3p |  |  |  |
|  | hsa-miR-31 |  |  |  |
|  | hsa-miR-625 |  |  |  |
|  | hsa-miR-556-3p |  |  |  |
|  | hsa-miR-647 |  |  |  |
|  | hsa-miR-663 |  |  |  |
|  | hsa-miR-509-3-5p |  |  |  |
|  | hsa-miR-24 |  |  |  |
|  | hsa-miR-448 |  |  |  |
|  | hsa-miR-583 |  |  |  |
|  | hsa-miR-100 |  |  |  |
|  | hsa-miR-431 |  |  |  |
|  | hsa-let-7a |  |  |  |
|  | hsa-miR-558 |  |  |  |
|  | hsa-miR-586 |  |  |  |
|  | hsa-miR-593 |  |  |  |
|  | hsa-miR-525-5p |  |  |  |
|  | hsa-miR-548c-3p |  |  |  |
|  | hsa-miR-503 |  |  |  |
|  | hsa-miR-650 |  |  |  |
|  | hsa-miR-346 |  |  |  |
|  | hsa-miR-214 |  |  |  |
|  | hsa-miR-634 |  |  |  |
|  | hsa-miR-494 |  |  |  |
|  | hsa-miR-512-5p |  |  |  |
|  | hsa-miR-596 |  |  |  |
|  | hsa-miR-618 |  |  |  |
|  | hsa-miR-15b |  |  |  |
|  | hsa-miR-944 |  |  |  |
|  | hsa-miR-107 |  |  |  |
|  | hsa-miR-767-3p |  |  |  |
|  | hsa-miR-302b |  |  |  |
|  | hsa-let-7f |  |  |  |
|  | hsa-miR-215 |  |  |  |
|  | hsa-miR-150 |  |  |  |
